# Maternal, Perinatal and Neonatal Outcomes of Triplet Pregnancies According to Chorionicity: Our 15-Year Experience in a Tertiary-Level Center

**DOI:** 10.3390/jcm13061793

**Published:** 2024-03-20

**Authors:** Mireia Bernal Claverol, Irene Aracil Moreno, María Ruiz Minaya, María Fernández Muñoz, Zurine Raquel Reyes Angullo, Pablo González Navarro, Natalio García-Honduvilla, Miguel A. Ortega, Santiago García Tizón, María P. Pintado-Recarte, Juan A. de León-Luis

**Affiliations:** 1 Department of Public and Maternal and Child Health, School of Medicine, Complutense University of Madrid, 28040 Madrid, Spainirene.aracill@salud.madrid.org (I.A.M.); mruiz341060@salud.madrid.org (M.R.M.); maria_ferny2@hotmail.com (M.F.M.); zurine.reyes@gmail.com (Z.R.R.A.); gineteca@gmail.com (S.G.T.); ppintadorec@yahoo.es (M.P.P.-R.); jaleon@ucm.es (J.A.d.L.-L.); 2 Department of Obstetrics and Gynecology, University Hospital Gregorio Marañón, 28009 Madrid, Spain; 3 Health Research Institute Gregorio Marañón, Doctor Esquerdo, 46, 28009 Madrid, Spain; 4 Maternal and Infant Research Investigation Unit, Alonso Family Foundation (UDIMIFFA), 28009 Madrid, Spain; 5 Methodology and Biostatistics Unit, Gregorio Marañón Health Research Institute (IiSGM), 28009 Madrid, Spain; pablo.gonzalez@iisgm.com; 6 Department of Medicine and Medical Specialities, Faculty of Medicine and Health Sciences, University of Alcalá, 28801 Alcalá de Henares, Spain; natalio.garcia@uah.es; 7 Ramón y Cajal Institute of Sanitary Research (IRYCIS), 28034 Madrid, Spain

**Keywords:** triplet pregnancy, maternal morbidity, fetal morbidity, perinatal morbidity, neonatal morbidity, perinatal mortality, chorionicity, monochorionic

## Abstract

**Introduction**: The goal of this study was to evaluate the effect of chorionicity on maternal, fetal and neonatal morbidity and mortality in triplet pregnancies in our environment. **Methods**: A retrospective observational study was carried out on triplet pregnancies that were delivered in a tertiary center between 2006 and 2020. A total of 76 pregnant women, 228 fetuses and 226 live newborns were analyzed. Of these triplet pregnancies, half were non-trichorionic. We analyzed maternal characteristics and obstetric, fetal, perinatal and neonatal complications based on their chorionicity, comparing trichorionic vs. non-trichorionic triplet pregnancies. Prematurity was defined as <34 weeks. We measured perinatal and neonatal mortality, composite neonatal morbidity and composite maternal morbidity. **Results**: Newborns with a monochorionic component had a lower gestational age at birth, presented greater prematurity under 34 weeks, lower birth weight, greater probability of birth weight under 2000 g and an APGAR score below 7 at 5 min after birth, more respiratory distress syndrome and, overall, higher composite neonatal morbidity. The monochorionic component of triple pregnancies may entail the development of complications intrinsic to shared circulation and require premature elective termination. This greater prematurity is also associated with a lower birth weight and to the main neonatal complications observed. These findings are in line with those that were previously published in the meta-analysis by our research group and previous literature. **Discussion**: Triplet gestations with a monochorionic component present a higher risk of obstetric, fetal and neonatal morbidity and mortality.

## 1. Introduction

Over the last three decades, social changes in relation to motherhood have promoted the use of assisted reproductive techniques (ART), thereby increasing the incidence of multiple pregnancies [1,2,3]. After the initial rise in the frequency of triplet pregnancies, its incidence began to decline and has currently stabilized [4,5,6,7,8] thanks to international recommendations to minimize the transfer of more than one or two embryos and the standardization of fetal reduction procedures [9,10]. Thus, at present, it is a rare type of pregnancy that requires highly complex multidisciplinary obstetrical and neonatal management in a tertiary referral center [11].

Triplet pregnancies are considered high-risk pregnancies due to the associated maternal, fetal, perinatal and neonatal morbidity, compared with twin and singleton pregnancies. In fact, the rates of preterm birth, premature rupture of membranes, restricted intrauterine growth, prematurity and low birth weight are notably high, as well as those of neonatal complications associated with prematurity [12,13,14,15,16,17]. In our setting, Revello published a study in 2019 describing the prevalence of maternal and obstetric complications in triplet pregnancies from their experience in another tertiary hospital in our city, where the most frequently observed obstetric complication in triplet pregnancies overall is the threat of preterm labor (56%), premature rupture of membranes (28.9%) and preeclampsia (20.4%) [18]. Similar findings regarding premature labor and pregnancy-induced hypertensive states have been described in studies of other countries and settings [12,18,19,20,21,22,23,24,25,26,27,28,29,30,31,32,33,34,35,36,37,38].

One of the main determinants of the morbidity associated with triplet pregnancy is chorionicity or the number of placental masses in the pregnancy. This could be due to the presence of shared circulation and vascular anastomosis in cases of pregnancies with monochorionic components (monochorionic and dichorionic triplet pregnancies) and the complications that may arise, such as twin-to-twin transfusion syndromes (TTTS) or selective intrauterine growth restriction (sIUGR). In fact, neonatal mortality was found to be mediated by the presence or absence of TTTS; survival was worse in dichorionic triplets with TTTS, but similar among dichorionic triplets without TTTS and trichorionic pregnancies [22].

However, chorionicity in triplet pregnancies has been associated with worse perinatal and neonatal outcomes, and it seems to affect neonatal survival and morbidity regardless of the presence of TTTS. Indeed, pregnancies with a monochorionic component have been reported to have a higher risk of prematurity and lower birth weight, ultimately leading to perinatal death. For instance, Kawaguchi found that monochorionic triplet pregnancies had a 2.6-fold greater risk of perinatal death compared with trichorionic triplet pregnancies [12]. In Bajoria’s study, this increased to an 8-fold risk, and this was mainly due to the higher risk of very low birth weight and prematurity at <30 weeks of gestation [14]. Geipel found that in monochorionic and dichorionic triplets compared to trichorionic triplets, there was a higher rate of intrauterine fetal death (8.8% vs. 1.5%) and of delivery at <32 weeks of gestation (47.4% vs. 32.2%) [39]. Indeed, Peress described that dichorionic triplets delivered on average two weeks earlier than trichorionic triplets [40]. In another study, Lopes Perdigao described that dichorionic triplet pregnancies were more likely to deliver before 30 weeks (25.5% vs. 8.3%) and to have very low birthweight neonates (41.8% vs. 22.2%) [22]. Maia described an increased intrauterine death in non-trichorionic triplets (8.8% vs. 1.5%) and increased perinatal mortality (5.5- vs. 8-fold higher). In their study, gestational age at delivery was found to be the main predictor of having no survivors from a triplet pregnancy, whereas preterm delivery together with the premature rupture of membranes and TTTS were found to be the main predictors of the number of children alive at the moment of hospital discharge [41].

In alignment with the observed higher rates of prematurity, low birth weight and perinatal death, some authors have also described higher rates of neonatal complications in non-trichorionic triplets. In 2017, Downing noted that non-trichorionic triplets, when compared to trichorionic triplets, had not only a significantly lower birth weight but also an increased composite fetal and neonatal morbidity (intrauterine growth restriction, respiratory distress syndrome, intraventricular hemorrhage and necrotizing enterocolitis), in addition to an increased risk of perinatal mortality [37]. Adegbite’s study also aligns with previous literature, showing that dichorionic triplets had a 5.5-fold higher risk of perinatal mortality, as well as a higher risk of delivery at <30 weeks and a birth weight of <1000 g. In addition, they observed higher rates of respiratory distress syndrome and intraventricular hemorrhage [42]. In another study, Spencer not only showed a lower gestational age at delivery and lower mean birth weights in dichorionic triplets but also a longer stay at the neonatal intensive care unit and higher rates of intubation and neonatal sepsis [43]. A recent meta-analysis conducted by Curado et al. found that dichorionic triamniotic triplets had worse perinatal outcomes than trichorionic triplets, with dichorionic triplets having a 5-fold higher risk of neurological morbidity, but no differences were found in composite respiratory and infectious morbidity [17].

To address the global effect of chorionicity on the morbidity and mortality of triplet pregnancies, our research group recently carried out a systematic review and meta-analysis of the literature published during the last 15 years and observed that non-trichorionic pregnancies had a higher risk of admission to the NICU, respiratory distress, sepsis, necrotizing enterocolitis and perinatal and intrauterine mortality [16]. This systematic review and meta-analysis included multiple hospitals in various countries and continents to obtain a sample that would allow us to observe significant associations among variables in such a rare obstetric event with such an impact on morbidity and mortality. Nevertheless, recognizing the importance of contextualizing these overall findings with the specific realities faced by different settings on a daily basis, we conducted this retrospective study in our center. This study focuses on analyzing the correlation between a range of maternal, fetal, perinatal and neonatal variables and maternal, fetal, perinatal and neonatal morbidity and mortality in a set of triplet pregnancies, including a comparative analysis of these variables according to chorionicity.

## 2. Materials and Methods

### 2.1. Sample

This was a retrospective observational study based on a hospital cohort of patients with triplet pregnancies who gave birth in our center between January 2006 and December 2020. Our center is a tertiary hospital in the capital city of Spain, Madrid, considered a reference center for obstetric care and high-risk and multiple pregnancies, and it is among the five biggest maternity wards in our country. The objective was to determine the prevalence of triplet pregnancies and associated maternal, fetal, perinatal and neonatal complications in our setting. To collect the data, a perinatal database based on the medical records of a tertiary hospital in Madrid, Spain, was reviewed over a period of 15 years. Initial triplet pregnancies in which first- or second-trimester abortions, miscarriages or fetal reductions occurred were not included in the study.

### 2.2. Variables

For all cases, we analyzed parameters related to maternal baseline characteristics, variables related to conception and pregnancy, obstetric variables, and variables concerning perinatal and neonatal outcomes.

Regarding the maternal baseline characteristics, the following were collected: maternal date of birth; maternal age, race and ethnicity (Caucasian, Hispanic, Asian, Arab and other); and past medical history (pregestational diabetes; thyroid, rheumatological, hematological, cardiovascular, or renal conditions; and endometriosis). Data on the method of conception (spontaneous or following assisted reproductive techniques), chorionicity (mono-, di- or trichorionic), parity, risk index for first-trimester chromosomal abnormalities or trisomies (low, intermediate, high or unknown) and the performance of invasive tests were also recorded, as well as the presence of anemia during pregnancy (defined as hemoglobin levels under 11 g/dL). Triplet pregnancies were diagnosed and chorionicity was established by ultrasound scan before 14 weeks of pregnancy, determining the number of placental masses, the presence of amniotic membrane(s) and membrane thickness and the presence of the lambda or T-sign. Triplet pregnancies were categorized into the following: monochorionic pregnancies, if the three fetuses shared one placenta and the T-sign could be seen among all fetuses; dichorionic pregnancies, if one set of monochorionic triplets sharing one placenta could be identified with the T-sign between each other, and the other fetus was seen to have its own placenta with the lambda sign visible; and trichorionic triplet pregnancies, if the three fetuses had a placenta of their own and, hence, the lambda sign could be seen among each other.

Regarding obstetric complications, data on the diagnosis of fetal abnormalities, such as malformations, growth disorders, such as macrosomia, restricted intrauterine growth (RIUG) or small for gestational age (SGA), and amniotic fluid disorders (oligo or polyhydramnios) were collected. Hypertensive states of pregnancy, including pre-eclampsia, eclampsia, gestational or pregestational hypertension (HT), and gestational diabetes, were also identified. Admissions for threatened preterm labor (TPL) requiring tocolytic treatment, therapeutic lung maturation, preterm premature rupture of membranes (PPROM), chorioamnionitis and intrahepatic cholestasis (IHC) were recorded.

Concerning the perinatal period, variables related to the mode of delivery (vaginal or cesarean section) and the following complications were registered: intrapartum fever, shoulder dystocia, postpartum hemorrhage and blood transfusion.

Finally, in the neonatal period, data on the following parameters were collected: sex of the newborn, birth weight in grams, vitality and neonatal resuscitation (NR), where level I is defined by the aspiration of secretions at birth, level II by use of oxygen through a nasal cannula, level III by use of positive airway pressure, level IV by airway intubation, and level V by cardiac massage or medication. Levels of umbilical cord pH at birth and APGAR score (1 and 5 min after birth), gestational age at birth, intrapartum or neonatal mortality and prematurity (<28 weeks, <32 weeks, <34 weeks, <37 weeks) were also collected. Complications included admission to the neonatal intensive care unit (NICU), hyaline membrane disease (HMD), respiratory distress syndrome (RDS), necrotizing enterocolitis (NEC), retinopathy of prematurity (ROP), severe intraventricular hemorrhage (IVH) grades III or IV and neonatal sepsis.

Various composite variables were constructed for multivariate regression analysis. The neonatal morbidity variable was defined as the occurrence of any of the following complications: NR ≥ 3, pH < 7.10, APGAR 5 min < 7, prematurity <32 weeks, EMH, NEC, ROP, HIV III–IV, sepsis or RDS. Pregestational comorbidity was defined as the occurrence of pregestational diabetes, endometriosis or rheumatologic, hematologic, cardiovascular, renal and thyroid disorders. Obstetric maternal morbidity was defined as the occurrence of IHC, chorioamnionitis, TPL, PPROM, preeclampsia, gestational HT or pregestational HT. Growth defects were defined as restricted intrauterine growth (RIUG) or small for gestational age (SGA). Hypertensive disorders of pregnancy were defined as gestational hypertension, preeclampsia and eclampsia.

### 2.3. Study Design and Data Analysis

#### 2.3.1. Descriptive Data

The initial analysis of descriptive data included all of the demographic, maternal, obstetric, fetal and neonatal variables that have been listed in the previous paragraphs. The analysis of descriptive data was carried out according to the type of variables measured. Quantitative variables are presented as median and interquartile range, and qualitative variables are presented as absolute and relative frequencies. The association between the quantitative variables of the study and chorionicity (trichorionic triplet pregnancies vs. non-trichorionic triplet pregnancies) was studied using the Wilcoxon signed-rank test. The association between qualitative variables and chorionicity was analyzed using Pearson’s chi-square test or Fisher’s exact test, as appropriate.

#### 2.3.2. Univariate Regression Analysis

Second, a univariate regression analysis based on chorionicity (non-trichorionicity vs. trichorionicity) was conducted with all the maternal, fetal, perinatal and neonatal variables mentioned. For the variables that did not show significant differences but that the authors considered to be clinically relevant, we constructed composite variables that were then compared between trichorionic and non-trichorionic pregnancies. This was the case for most of the variables included in the composite neonatal morbidity (NR > 3, pH < 7.10, prematurity <32 weeks, EMH, NEC, ROP, HIV III–IV and sepsis) and for all the variables included in the composite obstetric maternal morbidity (IHC, chorioamnionitis, TPL, PPROM, preeclampsia, gestational HT or pregestational HT). For the univariate regression analysis, the unit of the newborn’s weight was changed from grams to hectograms (dividing the newborn’s weight by 100) to interpret the results as the variation by 100 g of the newborn’s weight.

#### 2.3.3. Multivariate Regression Analysis

As it has been established in previous literature, prematurity, low birth weight and neonatal mortality were the most relevant complications in non-trichorionic triplet pregnancies when compared to trichorionic. Therefore, we decided to conduct several multivariate logistic regressions to deepen our understanding of these variables. The relationship between each of the complications (neonatal mortality, prematurity <34 weeks, prematurity <28 weeks, restricted intrauterine growth, small for gestational age, birth weight and neonatal morbidity) and a set of multiple explanatory variables was evaluated by multivariate logistic regression. The selection of the set of explanatory variables for each complication was carried out by minimizing the Akaike information criterion and following clinical criteria. The effect size is reported as the odds ratio together with its confidence interval.

For the following variables, the occurrence of the complication was not enough for a multivariate approach: neonatal mortality, prematurity <28 weeks, restricted intrauterine growth and small for gestational age. The univariate logistic regression model with adjustments for complications and explanatory variables conducted did not add relevant information.

For all the hypothesis tests performed, *p* < 0.05 was considered to indicate a statistically significant finding. All statistical analyses were performed with R Statistical Software (version 4.2.1; R Foundation for Statistical Computing, Vienna, Austria).

The study protocol was approved by the Ethics Committee for Medical Research on 12 November 2020, with the code OBS 04012020. The study data were stored in a database created for this purpose until the statistical analysis was performed.

## 3. Results

From a hospital cohort of 96,784 live newborns in our center between 2006 and 2020, a total of 76 pregnant women and 228 fetuses resulting in 226 live newborns were analyzed. This corresponds to 0.06% of all pregnancies and 0.24% of all live newborns (with a rate of 59.01 triplet pregnancies per 100,000 pregnancies and 235.58 newborns from triplet pregnancies per 100,000 newborns). Of these triplet pregnancies, a total of 35 (48.68%) were non-trichorionic.

### 3.1. Maternal and Demographic Characteristics and Obstetric and Fetal Complications

The maternal and demographic characteristics of the 76 pregnant women are presented in Table 1A. Three-quarters of the pregnancies were conceived by assisted reproductive technology, with the most frequently used form being IVF. Regarding the chorionicity of the pregnancies in our sample, 51.32% of them were trichorionic, 35.53% were dichorionic, and 10.53% were monochorionic. The obstetric and fetal complications in our sample, as well as between the study groups, are presented in Table 1B,C. None of the variables studied showed significant differences between the groups.

### 3.2. Perinatal and Neonatal Complications

The descriptive analysis of perinatal and neonatal complications in our sample and their comparison between non-trichorionic and trichorionic pregnancies is presented in Table 2.

**Figure 1:**
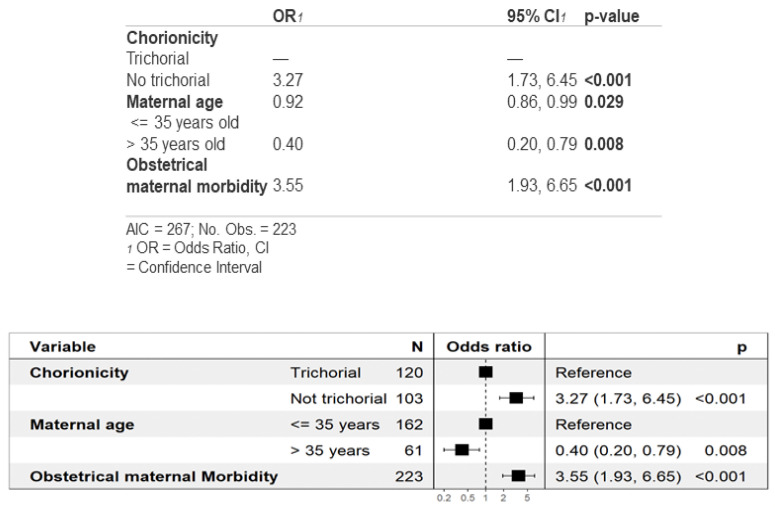
Multivariate analysis of prematurity before 34 weeks of gestation.

As shown in Table 2A, the analysis of gestational age and prematurity showed that non-trichorionic triplets were born at a lower gestational age on average, being more likely to be born before 34 weeks than trichorionic triplets. Figure 1 delves into this finding, showing how in the multivariate analysis for prematurity before 34 weeks, non-trichorionicity and maternal morbidity were found to be risk factors, whereas maternal age >35 years was found to act as a protective factor.

The mode of delivery was by cesarean section in practically all cases, as shown in Table 2B. There were no significant differences in the study variables regarding chorionicity. However, postpartum hemorrhage and blood transfusion requirements occurred exclusively in trichorionic pregnancies.

**Figure 2:**
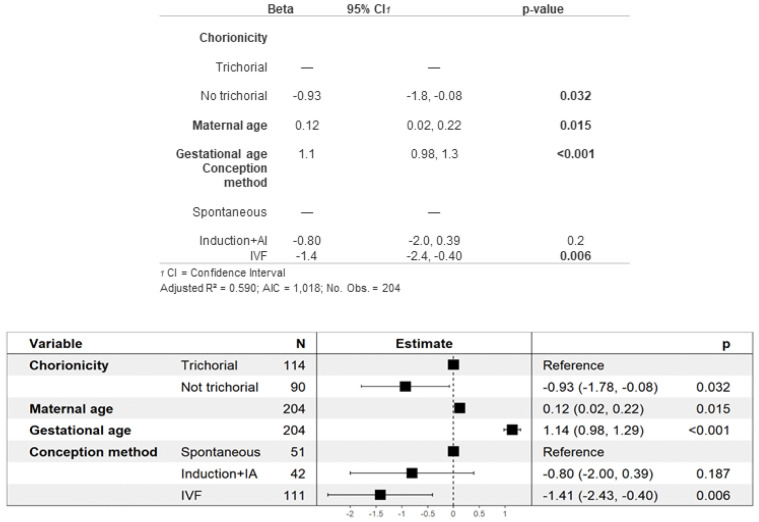
Multivariate analysis of newborn weight.

Neonatal complications are described in Table 2C. Specifically, the average birth weight was 1612 g, which was significantly higher in the trichorional group than the non-trichorional group. To analyze this finding in detail, we performed a multivariate analysis for the estimation of the mean birth weight, shown in Figure 2. Non-trichorionicity and IVF were found to be risk factors for lower birth weight, while maternal age and gestational age were associated with higher birth weight. As shown in Table 2C,D, in which neonatal complications and perinatal mortality are described, although 99% of the neonates survived the neonatal period, a total of 11 (4.8%) cases of neonatal death occurred, all of them in trichorionic pregnancies. Of those who survived the neonatal period, 26% required major neonatal resuscitation ≥III), and two of them obtained a cord pH under 7.10, both of which were from non-trichorionic pregnancies. A total of 9.6% of the newborns of non-trichorionic pregnancies obtained a 5 min APGAR score < 7, in contrast to 3.3% of the newborns from trichorionic pregnancies. A total of 34% of the newborns were admitted to the NICU, although the differences according to chorionicity were not significant. Regarding the main neonatal complication, 61% of the newborns presented respiratory distress syndrome, which was more frequent among the newborns of non-trichorionic pregnancies (71% vs. 53%). However, there were no significant differences in relation to chorionicity for the other neonatal complications.

**Figure 3:**
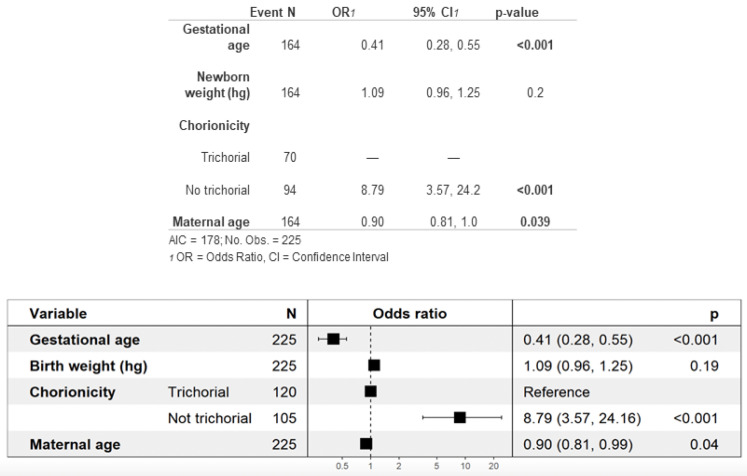
Multivariate analysis of neonatal morbidity.

Overall, a higher neonatal morbidity was found among newborns of non-trichorionic pregnancies than among those of trichorionic pregnancies (90% vs. 57%). As shown in Figure 3, the multivariate analysis for neonatal morbidity showed that newborns of non-trichorionic pregnancies have an 8.79 times higher risk of developing some type of morbidity than newborns of trichorionic pregnancies. Furthermore, the lower the gestational and maternal ages are, the higher the risk of neonatal comorbidity will be.

## 4. Discussion

### 4.1. Main Findings

In the last 15 years, our center delivered 96,784 newborns, of which 228 fetuses and 226 live newborns came from 76 triplet pregnancies, representing 0.24% of newborns. Taking into account the data of the Spanish National Statistics Institute for 2017 [8], the prevalence of triplet pregnancies in our center was more than triple the global one at the national level (77.15 vs. 24 per 100,000 deliveries), which identifies our hospital as a reference center for multiple pregnancies.

This study shows that triplet pregnancies are a rare obstetric event with high maternal, fetal and neonatal morbidity and mortality. In the analysis of our data based on chorionicity, we found that newborns with a monochorionic component had a lower gestational age at birth, a higher rate of prematurity before 34 weeks, a lower birth weight, a greater probability of birth weight under 2000 g and a 5 min APGAR score < 7, a higher rate of respiratory distress syndrome and, globally, higher composite neonatal morbidity, which was almost 9 times higher among non-trichorionic newborns. On one hand, non-trichorionic pregnancies had a higher risk of prematurity under 34 weeks when maternal age was under 35 years and in the presence of maternal morbidity. On the other hand, newborns from non-trichorionic pregnancies and those from ART had lower birth weights.

Finally, we compare our findings with those previously reported in the literature. Given the heterogeneity of the published data, the discrete sample size of the studies and the heterogeneity in the definitions of each variable, our main reference will be the meta-analysis that was recently carried out by our research team [16], indicating the articles included in it for the study of each of the variables to be discussed. In addition, we will also highlight the article by Revello et al. [19], included in the previous meta-analysis, because it is a retrospective study in another tertiary reference center in our city, whose data cover a period of time somewhat earlier than ours (2000–2010) and a larger cohort (147 triplets from 104,656 deliveries, prevalence of 140.5/100,000).

### 4.2. Assisted Reproductive Techniques, Demographic and Maternal Characteristics and Obstetric and Fetal Complications (Table 1)

#### 4.2.1. Prevalence of Triplet Pregnancies and Assisted Reproductive Technologies

In recent decades, a decrease in the number of triplet pregnancies has been observed. Live newborns of triplet pregnancies have gone from representing 471.03 per 100,000 live births in 2007 at the time of maximum incidence, to 117.26 per 100,000 live births in 2020. This may be related to the increased regulation of assisted reproductive technology (ART) in our setting, the minimization of transfers of more than one or two embryos, and the standardization of intrauterine surgery and fetal reduction procedures. Even so, three-quarters of our triplet pregnancies were conceived by ART, similar to other published series [16]. Although no significant differences were found based on chorionicity, a tendency was observed for monochorionic pregnancies to be twice as likely to come from spontaneous pregnancies compared to trichorionic and dichorionic pregnancies (50 vs. 26% vs. 17%). These data are consistent with those reported by Fennessy et al., who found that after the use of ART, the resulting triplet pregnancy was four times more likely to be trichorionic [20]. The fact that ART increases the risk of multiple pregnancies is largely attributed to the transfer of more than one embryo, which can result in trichorionic pregnancy but does not affect the probability of the spontaneous division of a single embryo, which would lead to a monochorionic pregnancy [21]. On the other hand, embryo transfer in the blastocyst phase, an increasingly common practice recommended in clinical practice guidelines, seems to be related to a higher risk of monochorionicity when dividing beyond the fifth or sixth day of culture [22]. Our sample did not achieve a significant difference, either because of an insufficient sample size or because of a significant decrease in the transfers of more than one embryo during the studied period.

#### 4.2.2. Demographic Maternal Characteristics and Obstetric and Fetal Complications

Our sample consisted mainly of young patients, with 18% of women presenting with pregestational comorbidities, including pregestational diabetes, thyroid, hematological and renal disorders, as well as endometriosis. There were no cases of cardiovascular or rheumatological diseases. The ethnic diversity of our sample, with 17% of the pregnant women being of non-Caucasian descent, facilitates the generalization of our findings to the general population of our setting in a large cosmopolitan city.

The main obstetric complication in this study, as well as in Revello’s study, was the threat of preterm labor (TPL), although in both cases, it was more than double the value reported in the meta-analysis (44% and 56% vs. 26.39%) [16,19], which included 25 studies [12,19,20,23,24,25,26,27,28,29,30,31,32,33,34,35,36,37,38,39,40,41,42,43,44]. The precise causes underlying these findings remain unclear, although they could be explained by the variability of the diagnostic criteria in the cut-off points of sonographic cervical length, differences in access to a referral hospital and the bias corresponding to a high-risk population [19]. There were no cases of maternal death.

The prevalence of hypertensive states of pregnancy in our study was similar to that of Revello and that of our meta-analysis (16.3% vs. 20.4% vs. 14.18%) [16,19]. Nevertheless, as emphasized in the meta-analysis, there is a notable variability (ranging between 3 and 59%) among the 34 included studies, which could potentially be attributed to variations in the definitions used for diagnosis [12,14,19,20,23,24,25,26,27,28,29,30,31,33,34,35,36,38,39,40,41,42,43,44,45,46,47,48,49,50,51,52,53,54]. Preeclampsia occurs in 20–45% of women with multiple pregnancies and 5% of women with singleton pregnancies [55,56,57,58], and there is an established correlation between its severity and the degree of multiple pregnancies [59]. This increased risk can also be attributed to the fact that women with triplet pregnancies are predominantly nulliparous and often conceive through ART, both of which are well-established independent risk factors for preeclampsia.

The proportion of fetal malformations in our study was remarkably lower than that reported in our meta-analysis (1.8% vs. 6.54%) [16], which included data from 17 studies [12,14,18,20,23,26,28,31,35,36,40,41,43,45,46,47,60]. This difference may arise from the fact that our series only included pregnancies resulting in triplet births, without considering the initial triplet pregnancies where fetal malformations were detected, leading to voluntary terminations of pregnancy or selective fetal reductions.

There were also fewer growth defects compared to those reported in our meta-analysis (13% for IUGR and 6.3% for SGA vs. 34.92% for IUGR/SGA) [16], which included data from 19 studies [18,20,25,26,28,29,30,33,34,35,36,39,41,43,44,50,53,54,60]. Despite the fact that the use of specific triplet growth tables instead of universal ones is still controversial, it is a generalized conception that triplet pregnancies should be considered high-risk pregnancies for SGA and IUGR. Hence, it might be possible that the use of different growth tables that might be more or less suitable for a specific population or subpopulation (such as triplet pregnancies), could account for a certain level of heterogeneity in the identification of growth defects in previously published studies.

We need to take into consideration that more than half (65%) of the triplet pregnancies in our sample experienced obstetric complications. Therefore, it is crucial to provide close monitoring and follow-up, prioritizing screening, early diagnosis, prevention and the management of both threat of preterm labor and hypertensive states of pregnancy. However, after comparatively analyzing our data based on chorionicity, we did not find significant differences in the development of obstetric or fetal complications between the groups, so it is possible that the increased risk observed in triplet pregnancy compared to twin pregnancy is due to the number of fetuses rather than chorionicity.

### 4.3. Perinatal and Neonatal Complications 

#### 4.3.1. Prematurity

The main perinatal complication observed in our study was preterm delivery. In our sample, the proportion of deliveries occurring before 34 weeks was comparable to that reported in our meta-analysis (65% vs. 57.58%) [16], which included 9 articles [12,23,24,25,29,36,48,53,61]. However, there was a notable difference in the incidence of births occurring before 32 weeks, with a lower percentage observed in our study compared to that in the meta-analysis (28% vs. 40.94%) [16], which included 18 studies [18,19,20,24,26,28,31,36,43,45,46,52,53,60,61,62,63,64]. Notably, our study revealed a proportion of births occurring before 28 weeks that closely resembled the findings from Revello’s study, although it was only half of what was reported in our meta-analysis (6.7% vs. 7.5% vs. 12.92%) [16,19], which included 17 studies [12,18,19,20,23,25,26,29,31,32,36,43,44,45,46,53,64]. This trend toward less pronounced prematurity in triplet pregnancies in our setting is not entirely surprising. While we have described that a substantial proportion of obstetric and fetal complications that may require the early termination of pregnancy, it is presently recommended to electively terminate triplet pregnancies around 35–36 weeks. This widely accepted recommendation [65], originating from the study conducted by Khan et al. in 2003 [66], in which they observed a higher occurrence of stillbirths compared to neonatal deaths beyond weeks 35–36 [67].

In terms of the impact of chorionicity on prematurity, non-trichorionic pregnancies have a higher probability of preterm birth before 34 weeks, while trichorionic pregnancies exhibit a notably higher number of newborns delivered between 34 and 36 + 6 weeks. This disparity could be attributed to the termination of pregnancies affected by intrinsic complications associated with monochorionic placentas, such as twin-to-twin transfusion syndrome or knots in the umbilical cords [68,69]. Our meta-analysis indicated that trichorionic triplet pregnancies demonstrated a lower risk of prematurity before 37 weeks [OR = 0.51 95%, CI (0.25, 1.00)] and before 32 weeks [OR = 0.56 95%, CI (0.31, 1.00)], although these differences did not reach statistical significance [16]. These findings align with some of the studies included in our meta-analysis [12,18,31,43,48]. It is important to note that the majority of triplet pregnancies result in premature births occurring around 30–32 weeks, with non-trichorionic pregnancies being only 1 to 3 weeks more premature than trichorionic pregnancies [14,18,47]. Thus, it is not surprising that substantial differences in overall prematurity or prematurity before 32 weeks based on chorionicity were not observed.

Finally, pregnant women with obstetric maternal morbidity face a higher risk of giving birth before 34 weeks (Figure 1). This observation seems reasonable since conditions like hypertensive states of pregnancy, intrahepatic cholestasis, preterm ruptured membranes or the threat of preterm labor may lead to the early termination of pregnancy. Furthermore, newborns from non-trichorionic pregnancies are more likely to be born before 34 weeks due to their increased probability of fetal complications that may result in early termination and preterm birth. The reason why advanced maternal age (>35 years) acts as a protective factor against prematurity remains to be elucidated, although this finding has been previously reported in the literature [70].

#### 4.3.2. Delivery and Immediate Postpartum Period

Regarding the mode of delivery, most of our patients underwent cesarean section, since this is the current recommendation in clinical practice guidelines regardless of the chorionicity of the pregnancy [24]. The proportion of postpartum hemorrhages was found to be half of what was described by Revello, but similar to the figures reported in our meta-analysis (5.3% vs. 15.6% vs. 4.58%) [16,19], which included seven studies [26,27,33,34,35,45,71]. This variation may be attributed to changes in protocols for managing postpartum hemorrhage in recent years, promoting more proactive medical management and prevention, and resulting in reduced interventionism. It is noteworthy that, in our study, postpartum hemorrhages were only observed in trichorionic pregnancies. This could be due to the increased number of placentas, which potentially increases the risk of placental implantation defects and associated hemorrhage. However, the authors are not aware of any publications that study the relationship between chorionicity and postpartum hemorrhage in triplet pregnancies. In twin pregnancies, chorionicity does not seem to affect the risk of developing postpartum hemorrhage [72].

### 4.4. Neonatal Complications

The neonatal data are consistent with previous publications: newborns with a monochorionic component tend to have lower birth weights, poorer APGAR scores, higher rates of respiratory distress syndrome and overall increased neonatal comorbidity.

#### 4.4.1. Birth Weight

The mean birth weight of the newborns in our sample is similar to the mean birth weight reported in our meta-analysis (1612 g vs. 1638 g) [16], which comprised 36 studies [12,14,18,20,23,24,26,27,30,31,32,33,34,35,36,37,39,40,41,42,43,44,45,46,47,48,50,54,60,62,63,64,71,73,74,75,76]. Regarding the impact of chorionicity on birth weight, newborns from trichorionic pregnancies weighed significantly more than those from non-trichorionic ones, in line with findings from previous studies, and this is probably related to the higher prevalence of prematurity.

In our sample, a notable association between birth weight and chorionicity was observed, with newborns from non-trichorionic pregnancies having a higher probability of weighing less than 2000 g at birth, which is a clinically relevant threshold for monitoring and NICU admission. Although statistical significance was not reached, a similar trend was observed for very low birth weight (<1500 g), consistent with findings from previous publications [12,48,77].

Finally, our analysis revealed several factors influencing birth weight. Newborns of mothers of advanced age and those with a higher gestational age exhibited higher birth weights. Conversely, those from non-trichorionic pregnancies and pregnancies achieved by IVF had lower birth weights (Figure 2). Interestingly, this multivariate analysis of birth weight was not previously examined in our group’s meta-analysis [16], nor in the study by Revello et al. [19], despite the existence of relevant data published in the literature for each of these factors. Moreover, the observed direct relationship between gestational age and birth weight is expected and seems reasonable. Probably for this same reason, newborns from non-trichorionic pregnancies, which tend to be more premature, also have lower birth weights, as previously reported by Kawaguchi, Morikawa and Lamb et al. [12,48,77]. However, studies by Fennessy and Morency et al., the most relevant and recent investigations on the impact of ART on triplet pregnancies outcomes, did not find significant differences in neonatal weight based on the use of IVF [20,26]. A possible explanation for our findings could be that in our sample, IVF pregnancies were associated with alterations in placentation, which, in turn, could be related to growth defects and lower birth weight. Finally, the relationship between advanced maternal age and higher birth weight has already been described by Salihu et al. [74], although the underlying pathophysiological explanation remains to be clarified.

#### 4.4.2. APGAR Scores, NICU Admission and Neonatal Complications

The percentage of APGAR scores below 7 at 5 min in triplet pregnancies was similar to that observed in our meta-analysis (6.2% vs. 8.38%) [16]. Our data show that neonates born from non-trichorionic pregnancies, which generally have higher neonatal morbidity rates, also tend to have worse APGAR scores. However, we have evidence from only two previous studies that analyzed the impact of chorionicity on the percentage of 5 min APGAR scores below 7, and no significant differences were observed in those studies [12,50].

In our sample, no significant differences were observed in terms of chorionicity and admission to the NICU, unlike previous studies that reported a higher frequency of NICU admissions for newborns from non-trichorionic pregnancies [12,42,43]. However, it should be noted that the total number of admissions in our sample was significantly lower compared to what was reported in our meta-analysis (31% vs. 78.69%), which included 18 studies [12,16,23,26,27,28,29,30,35,36,37,40,41,42,43,45,50,52,78]. The extent to which improvements in maternal and perinatal management, as well as in neonatal care for triplet pregnancies, have influenced the criteria for NICU admission remains uncertain.

Respiratory distress syndrome emerged as the predominant neonatal complication, presenting significant differences based on chorionicity, with higher prevalence among non-trichorionic neonates (71% vs. 53%), consistent with previous literature [12,14,43,47,50].

Moreover, it is noteworthy that our sample demonstrated a higher percentage of neonatal sepsis compared to our previous meta-analysis (26% vs. 5.48%) [16], which encompassed 17 studies [12,14,23,26,27,28,29,30,31,35,36,40,44,47,50,73,78]. This observation could potentially be associated with the increased frequency of the premature rupture of membranes in our sample. Similarly, severe intraventricular hemorrhage was more prevalent in our current study compared to our previous meta-analysis (12 vs. 4.65%) [16], which included 25 studies [12,20,23,26,27,29,30,31,32,33,34,36,39,43,44,45,47,49,50,52,60,71,73,78].

No significant differences were observed in the occurrence of necrotizing enterocolitis or neonatal sepsis based on chorionicity, which contrasts with previous studies reporting an increased risk in non-trichorionic triplet pregnancies [12,23,30,31]. Both complications are more prevalent in preterm newborns, and it has already been described how the rate of prematurity is increased in triplet pregnancies, particularly those with a monochorionic component. The lack of statistical significance in our data is likely attributed to the limited sample size, which may have affected the power to detect significant associations.

#### 4.4.3. Neonatal Morbidity

In the multivariate analysis of neonatal morbidity, we identified a higher gestational age and an advanced maternal age as protective factors. One of the most notable findings of our analysis is the observation of an 8.79 times higher incidence of neonatal morbidity in non-trichorionic pregnancies (Figure 3). This result is consistent with the findings reported by the eight studies included in our previous meta-analysis [16], as both our sample and previous studies have demonstrated higher rates of prematurity and various associated neonatal complications in pregnancies with a monochorionic component [12,14,31,42,43,47,48,50].

#### 4.4.4. Neonatal Mortality

The noticeable increased risk of neonatal mortality in triplet pregnancies within our sample (48/1000 live births) stands in contrast to the rates observed in singleton pregnancies (1.18/1000 live births) and twin pregnancies (6.06/1000 live births) [79]. Our findings on neonatal mortality align with the results reported by the 34 studies included in our previous meta-analysis [12,14,18,19,20,23,25,26,28,29,31,32,33,34,35,36,37,39,41,43,44,45,46,47,48,49,52,60,61,62,64,71,73,78]. Interestingly, no significant differences in neonatal mortality were identified according to chorionicity. This could be attributed to the fact that, while newborns from non-trichorionic pregnancies may exhibit a higher prevalence of fetal and neonatal comorbidities, the increasing trend towards fetal interventionism in complications intrinsic to monochorionicity, such as twin-to-twin transfusion syndrome (TTTS), could explain the lack of a proportional increase in neonatal mortality.

As we previously emphasized, maternal complications may be more closely associated with the number of fetuses rather than chorionicity. Conversely, the number of placentas in a multiple pregnancy fundamentally affects the development of neonatal complications.

### 4.5. Strengths and Limitations

One of the main strengths of this study lies in the extensive range of variables assessed, encompassing obstetric, maternal, fetal, perinatal and neonatal factors. In addition, our sample population is ethnically diverse, and our data were collected from a public hospital with almost universal access to high-quality ART and obstetric and neonatal care. This comprehensive approach provides a holistic, multidisciplinary and up-to-date understanding of the reality of the morbidity and mortality associated with triplet pregnancies in our specific context. 

Another strength of this study is the detailed analysis of our data, allowing for meaningful comparisons with the results of the recently published meta-analysis conducted by our research group [16] and largely corroborating those findings. Despite the smaller sample size compared to the study by Revello et al. [19], our data enabled us to explore various aspects of maternal and perinatal morbidity beyond the neonatal complications analyzed by Revello et al. Furthermore, we conducted multivariate analyses on clinically relevant perinatal variables, expanding the scope of our study.

Our study has several limitations. First, its retrospective observational design restricts the quality of the evidence presented, and the results, being derived from a single-center study, may not be as generalizable as those obtained from multi-center databases. Additionally, the sample size is insufficient to obtain statistical significance in some of the analyses performed. Finally, given that our center is a referral hospital, some patients were referred to us after initiating follow-up at other facilities, leading to incomplete data for certain variables.

## 5. Conclusions

Triplet pregnancies are a rare obstetric event, representing a rate of 59.01 triplet pregnancies per 100,000 pregnancies based on 15 years of experience at our center. Triplet pregnancies are high-risk pregnancies associated with increased maternal morbidity and perinatal morbidity and mortality. Notably, this increased risk is particularly significant in newborns from non-trichorionic pregnancies, who face a higher risk of developing neonatal complications when compared to newborns from trichorionic pregnancies. These complications include lower birth weight, earlier gestational age at birth, poorer APGAR scores, and an increased risk of respiratory distress syndrome. Through our detailed analysis and discussion of the data, we have consistently observed findings that align with previous publications, reinforcing the understanding that chorionicity plays a significant role in perinatal and neonatal outcomes.

## Figures and Tables

**Table 1:** Maternal demographic characteristics obstetric and fetal complications.

	Chorionicity			
	Non-Trichorionic *N* = 351	Trichorionic *N* = 411	Total *N* = 761	*p*-Value
(A) Maternal and demographic characteristics (*N* = 76)				
Maternal age	34.0 (5.4)	32.5 (3.4)	33.2 (4.4)	0.122
Nulliparity	26/35 (74%)	33/41 (80%)	59/76 (78%)	0.54
Ethnicity				0.33
Caucasian	24/27 (89%)	20/26 (77%)	44/53 (83%)	
Hispanic	1/27 (3.7%)	5/26 (19%)	6/53 (11%)	
Arabic	2/27 (7.4%)	1/26 (3.8%)	3/53 (5.7%)	
Pre-gestational comorbidity	8/35 (23%)	6/41 (15%)	14/76 (18%)	0.44
Pregestational diabetes	1/35 (2.9%)	0/41 (0%)	1/76 (1.3%)	0.53
Thyroid disorders	3/35 (8.6%)	3/41 (7.3%)	6/76 (7.9%)	>0.93
Hematologic disorders	3/35 (8.6%)	1/41 (2.4%)	4/76 (5.3%)	0.33
Kidney disorders	0/35 (0%)	1/41 (2.4%)	1/76 (1.3%)	>0.93
Endometriosis	1/35 (2.9%)	2/41 (4.9%)	3/76 (3.9%)	>0.93
Conception method				0.114
Spontaneous	7/30 (23%)	10/38 (26%)	17/68 (25%)	
Induction + AI	3/30 (10%)	11/38 (29%)	14/68 (21%)	
IVF	20/30 (67%)	17/38 (45%)	37/68 (54%)	
High risk for trisomies	19/35 (54%)	20/41 (49%)	39/76 (51%)	0.64
Invasive testing performed	4/35 (11%)	7/41 (17%)	11/76 (14%)	0.54
Gestational anemia	16/29 (55%)	18/36 (50%)	34/65 (52%)	0.74
(B) Obstetric complications (*N* = 76)				
Oligoamnios	1/35 (2.9%)	3/40 (7.5%)	4/75 (5.3%)	0.63
Preeclampsia	3/35 (8.6%)	5/40 (12%)	8/75 (11%)	0.73
Eclampsia	0/35 (0%)	0/40 (0%)	0/75 (0%)	
Gestational hypertension	2/35 (5.7%)	2/40 (5.0%)	4/75 (5.3%)	>0.93
Pregestational hypertension	0/35 (0%)	1/40 (2.5%)	1/75 (1.3%)	>0.93
Threatened preterm labor (TPL)	12/35 (34%)	21/40 (52%)	33/75 (44%)	0.114
Lung maturation with corticosteroids	27/35 (77%)	31/40 (78%)	58/75 (77%)	>0.94
Gestational diabetes	5/33 (15%)	1/40 (2.5%)	6/73 (8.2%)	0.0853
Premature rupture of membranes (PROM)	4/35 (11%)	7/41 (17%)	11/76 (14%)	0.54
Chorioamnionitis	1/34 (2.9%)	3/41 (7.3%)	4/75 (5.3%)	0.63
Intrahepatic cholestasis (IHC)	3/35 (8.6%)	5/41 (12%)	8/76 (11%)	0.73
Twin-to-twin transfusion syndrome (TTTS)	1/35 (2.9%)	0/41 (0%)	1/76 (1.3%)	0.53
Composite Maternal Comorbidity	20/35 (57%)	27/41 (66%)	47/76 (62%)	0.44
(C) Fetal complications (*N* = 228)				
	**Chorionicity**			
	**Non-Trichorionic** ***N* = 105**	**Trichorionic** ***N* = 123**	**Total,** ***N* = 2281**	***p*-Value**
Malformation	3/105 (2.9%)	1/123 (0.8%)	4/228 (1.8%)	0.22
Intrauterine growth restriction (IUGR)	13/103 (13%)	16/123 (13%)	29/228 (13%)	>0.93
Small for gestational age (SGA)	4/103 (3.9%)	10/120 (8.3%)	14/223 (6.3%)	0.24
1 Mean (SD); *n*/*N* (%)				
2 Wilcoxon rank sum test				
3 Fisher’s exact test				
4 Pearson’s Chi-squared test				

**Table 2:** Perinatal and neonatal complications.

(A) Gestational age and prematurity (*N* = 228)				
Chorionicity				
	No trichorial, *N* = 1051	Trichorial, *N* = 1231	Total, *N* = 2281	*p*-Value
Gestational age at delivery (weeks)				**0.0143**
*N*	105	120	225	
Mean (SD)	31.83 (2.42)	32.35 (2.84)	32.11 (2.66)	
Prematurity < 34	78/105 (74%)	69/120 (57%)	147/225 (65%)	**0.0084**
Prematurity < 32	33/105 (31%)	30/120 (25%)	63/225 (28%)	0.34
Prematurity < 28	6/105 (5.7%)	9/120 (7.5%)	15/225 (6.7%)	0.64
Prematurity [34–36+6] weeks	30/105 (29%)	54/120 (45%)	84/225 (37%)	**0.0114**
Prematurity [32–33+6] weeks	42/105 (40%)	38/120 (32%)	80/225 (36%)	0.24
Prematurity [28–31+6]	24/105 (23%)	18/120 (15%)	42/225 (19%)	0.134
(B) Intrapartum complications (*N* = 76)				
	Chorionicity			
	Non-Trichorionic, *N* = 351	Trichorionic, *N* = 411	Total, *N* = 761	*p*-Value
Cesarean section	33/34 (97%)	39/40 (98%)	72/74 (97%)	>0.92
Cesarean indication				0.62
BPP	2/35 (5.7%)	6/41 (15%)	8/76 (11%)	
Elective/programmed	15/35 (43%)	20/41 (49%)	35/76 (46%)	
Spontaneous labor start	12/35 (34%)	10/41 (24%)	22/76 (29%)	
Maternal risk or disorder	4/35 (11%)	3/41 (7.3%)	7/76 (9.2%)	
Postpartum hemorrhage	0/35 (0%)	4/41 (9.8%)	4/76 (5.3%)	0.122
Transfusion of blood concentrates	0/35 (0%)	2/41 (4.9%)	2/76 (2.6%)	0.52
(C) Neonatal complications (*N* = 228)				
	Chorionicity			
	Non-trichorinic, *N* = 1051	Trichorionic, *N* = 1231	Total, *N* = 2281	*p*-Value
Newborn weight				**0.0053**
*N*	104	123	227	
Mean (SD)	1527 (412)	1686 (443)	1612 (435)	
Newborn weight < 1500 g	46/105 (44%)	39/123 (32%)	85/228 (37%)	0.0604
Newborn weight < 2000 g	95/105 (90%)	92/123 (75%)	187/228 (82%)	0.0024
Newborn weight < 2500 g	105/105 (100%)	122/123 (99%)	227/228 (100%)	>0.92
pH < 7.10	2/101 (2.0%)	0/114 (0%)	2/215 (0.9%)	0.22
Apgar 5 min < 7	10/104 (9.6%)	4/122 (3.3%)	14/226 (6.2%)	**0.0494**
REA ≥ 3	33/105 (31%)	27/123 (22%)	60/228 (26%)	0.114
Neonatal ICU admission	38/105 (36%)	32/123 (26%)	70/228 (31%)	0.104
NICU admission time	3.7 (10.1)	2.2 (6.9)	2.9 (8.5)	0.123
NICU admission time ≥ 7 d	15/105 (14%)	22/123 (18%)	37/228 (16%)	0.54
Hyaline membrane disease	32/105 (30%)	46/123 (37%)	78/228 (34%)	0.34
Necrotizing enterocolitis	12/105 (11%)	19/123 (15%)	31/228 (14%)	0.44
Retinopathy of prematurity	13/105 (12%)	23/123 (19%)	36/228 (16%)	0.24
Severe intraventricular hemorrhage	9/105 (8.6%)	19/123 (15%)	28/228 (12%)	0.114
Neonatal sepsis	25/105 (24%)	35/123 (28%)	60/228 (26%)	0.44
Respiratory distress syndrome	75/105 (71%)	65/123 (53%)	140/228 (61%)	**0.0044**
Composite neonatal morbidity	94/105 (90%)	70/123 (57%)	164/228 (72%)	**<0.0014**
(D) Perinatal mortality (*N* = 228)				
Intrapartum death	1/105 (1.0%)	1/123 (0.8%)	2/228 (0.9%)	>0.92
Neonatal death	6/105 (5.7%)	5/123 (4.1%)	11/228 (4.8%)	0.64
1 Mean (SD); *n*/*N* (%)				
2 Fisher’s exact test				
3 Wilcoxon rank sum test				
4 Pearson’s Chi-squared test				

## Data Availability

The datasets used and/or analyzed during the current study are available from the corresponding author upon reasonable request.

## References

[1] Strauss A., Winkler D., Middendorf K., Kümper C., Herber-Jonat S., Schulze A. (2008). Higher order multiples--socioeconomic impact on family life. Eur. J. Med. Res..

[2] Stone J., Kohari K.S. (2015). Higher-order Multiples. Clin. Obstet. Gynecol..

[3] Grantz K.L., Kawakita T., Lu Y.-L., Newman R., Berghella V., Caughey A., SMFM Research Committee (2019). SMFM Special Statement: State of the science on multifetal gestations: Unique considerations and importance. Am. J. Obstet. Gynecol..

[4] Martin J.A., Osterman M.J., Thoma M.E. (2016). Declines in Triplet and Higher-order Multiple Births in the United States, 1998–2014. NCHS Data Brief..

[5] Martin J.A., Hamilton B.E., Osterman M.J.K., Driscoll A.K. (2019). Births: Final Data for 2018. Natl. Vital Stat. Rep..

[6] Blondel B., Kaminski M. (2002). Trends in the occurrence, determinants, and consequences of multiple births. Semin. Perinatol..

[7] Multiple Births National Center for Health Statistics. Centers for Disease Control and Prevention. https://www.cdc.gov/nchs/fastats/multiple.htm.

[8] Numero de Nacimientos de Gestaciones Triples en España Durante el año INE. https://www.ine.es/jaxi/Datos.htm?tpx=50541.

[9] Morlando M., Ferrara L., D’Antonio F., Lawin-O’Brien A., Sankaran S., Pasupathy D., Khalil A., Papageorghiou A., Kyle P., Lees C. (2015). Dichorionic triplet pregnancies: Risk of miscarriage and severe preterm delivery with fetal reduction versus expectant management. Outcomes of a cohort study and systematic review. BJOG Int. J. Obstet. Gynaecol..

[10] Papageorghiou A.T., Avgidou K., Bakoulas V., Sebire N.J., Nicolaides K.H. (2006). Risks of miscarriage and early preterm birth in tricho-rionic triplet pregnancies with embryo reduction versus expectant management: New data and systematic review. Hum. Reprod. Oxf Engl..

[11] Mhatre M., Craigo S. (2021). Triplet pregnancy: What do we tell the prospective parents. Prenat. Diagn..

[12] Kawaguchi H., Ishii K., Yamamoto R., Hayashi S., Mitsuda N. (2013). Perinatal death of triplet pregnancies by chorionicity. Am. J. Obstet. Gynecol..

[13] Norwitz E.R., Edusa V., Park J.S. (2005). Maternal Physiology and Complications of Multiple Pregnancy. Semin. Perinatol..

[14] Bajoria R., Ward S.B., Adegbite A.L. (2006). Comparative study of perinatal outcome of dichorionic and trichorionic iatrogenic triplets. Am. J. Obstet. Gynecol..

[15] Wen S.W., Demissie K., Yang Q., Walker M.C. (2004). Maternal morbidity and obstetric complications in triplet pregnancies and quad-ruplet and higher-order multiple pregnancies. Am. J. Obstet. Gynecol..

[16] Bernal Claverol M., Ruiz Minaya M., Aracil Moreno I., Tizón S.G., Pintado Recarte P., Alvarez-Mon M., Arribas C.B., Ortega M.A., De Leon-Luis J.A. (2022). Maternal, Perinatal and Neonatal Outcomes of Triplet Pregnancies According to Chorionicity: A Systematic Review of the Literature and Me-ta-Analysis. J. Clin. Med..

[17] Curado J., D’Antonio F., Papageorghiou A.T., Bhide A., Thilaganathan B., Khalil A. (2019). Perinatal mortality and morbidity in triplet pregnancy according to chorionicity: Systematic review and meta-analysis. Ultrasound Obstet. Gynecol..

[18] Revello R., De la Calle M., Moreno E., Duyos I., Salas P., Zapardiel I. (2013). Maternal morbidity on 147 triplets: Single institution experience. J. Matern.-Fetal Neonatal Med..

[19] Fennessy K.M., Doyle L.W., Naud K., Reidy K., Umstad M.P. (2015). Triplet Pregnancy: Is the Mode of Conception Related to Perinatal Outcomes?. Twin Res. Hum. Genet..

[20] Kyeong K.S., Shim J.Y., Oh S.Y., Won H.S., Lee P.R., Kim A., Yun S.C., Kang P.N., Choi S.J., Roh C.R. (2019). How much have the perinatal outcomes of triplet pregnancies improved over the last two decades?. Obstet. Gynecol. Sci..

[21] Lappen J.R., Hackney D.N., Bailit J.L. (2016). Maternal and neonatal outcomes of attempted vaginal compared with planned cesarean delivery in triplet gestations. Am. J. Obstet. Gynecol..

[22] Perdigao J.L., Straub H., Zhou Y., Gonzalez A., Ismail M., Ouyang D.W. (2016). Perinatal and obstetric outcomes of dichorionic vs trichorionic triplet pregnancies. Am. J. Obstet. Gynecol..

[23] Morency A.M., Shah P.S., Seaward P.G.R., Whittle W., Murphy K.E. (2016). Obstetrical and neonatal outcomes of triplet births—*Spontaneous* versus assisted reproductive technology conception. J. Matern.-Fetal Neonatal Med..

[24] Peress D., Dude A., Peaceman A., Yee L.M. (2019). Maternal and neonatal outcomes in triplet gestations by trial of labor versus planned cesarean delivery. J. Matern.-Fetal Neonatal Med..

[25] Rajan P., Murki S., Vavilala S., Surubhotla N. (2018). Maternal and Early Perinatal Outcomes of Triplet Pregnancy: Study of 82 Triplets from a Single Perinatal Center in South India. J. Obstet. Gynecol. India.

[26] Sato Y., Ishii K., Yokouchi T., Murakoshi T., Kiyoshi K., Nakayama S., Yonetani N., Mitsuda N. (2016). Incidences of Feto-Fetal Transfusion Syndrome and Perinatal Outcomes in Triplet Gestations with Monochorionic Placentation. Fetal Diagn. Ther..

[27] Al-Sunaidi M.I., Al-Shahrani M.S. (2011). Fetomaternal and neonatal outcome of triplet pregnancy. Saudi Med. J..

[28] Simões T., Queiros A., Gonçalves M.R., Periquito I., Silva P., Blickstein I. (2015). Perinatal outcome of dichorionic-triamniotic as compared to trichorionic triplets. J. Perinat. Med..

[29] Arlettaz Mieth R., Ersfeld S., Douchet N., Wellmann S., Bucher H.U. (2011). Higher multiple births in Switzerland: Neonatal outcome and evolution over the last 20 years. Swiss Med. Wkly..

[30] Chibber R., Fouda M., Shishtawy W., Al-Dossary M., Al-Hijji J., Amen A., Mohammed A.T. (2013). Maternal and neonatal outcome in triplet, quad-ruplet and quintuplet gestations following ART: A 11-year study. Arch. Gynecol. Obstet..

[31] Mazhar S.B., Rahim F., Furukh T. (2008). Fetomaternal outcome in triplet pregnancy. J. Coll. Physicians Surg. Pak. JCPSP.

[32] Al-Suleiman S.A., Al-Jama F.E., Rahman J., Rahman M.S. (2006). Obstetric complications and perinatal outcome in triplet pregnancies. J. Obstet. Gynaecol..

[33] AlBasri S.F., Shouib G.M., Bajouh O.S., Nasrat H.A., Ahmad E., AlGreisi F.M. (2017). Maternal and neonatal outcomes in twin and triplet gestations in Western Saudi Arabia. Saudi Med. J..

[34] Luke B., Brown M.B. (2008). Maternal morbidity and infant death in twin vs triplet and quadruplet pregnancies. Am. J. Obstet. Gynecol..

[35] Moore E.S., Elnaggar A.C., Wareham J.A., Ramsey C.J., Sumners J.E. (2012). Neonatal functional lung maturity relative to gestational age at delivery, fetal growth, and pregnancy characteristics in triplet births. J. Matern.-Fetal Neonatal Med..

[36] Almeida P., Domingues A.P., Belo A., Fonseca E., Moura P. (2014). Triplet pregnancies: Perinatal outcome evolution. Rev. Bras. Ginecol. E Obs..

[37] Downing M., Sulo S., Parilla B. (2017). Perinatal and Neonatal Outcomes of Triplet Gestations Based on Chorionicity. Am. J. Perinatol. Rep..

[38] Kraemer B., Becker S., Kagan K.O., Hahn M., Rajab T.K., Wallwiener D., Kraemer E., Abele H. (2009). Twenty-six triplet pregnancies: A retrospective analysis. Arch. Gynecol. Obstet..

[39] Geipel A., Berg C., Katalinic A., Plath H., Hansmann M., Germer U., Gembruch U. (2005). Prenatal diagnosis and obstetric outcomes in triplet pregnancies in relation to chorionicity. BJOG Int. J. Obstet. Gynaecol..

[40] Peress D.A., Peaceman A.M., Yee L.M. (2017). Evaluation of Trichorionic versus Dichorionic Triplet Gestations from 2005 to 2016 in a Large, Referral Maternity Center. Am. J. Perinatol..

[41] Maia C.B., Liao A.W., Brizot M.L., Francisco R.P.V., Zugaib M. (2016). Prediction of perinatal mortality in triplet pregnancies. Arch. Gynecol. Obstet..

[42] Adegbite A.L., Ward S.B., Bajoria R. (2005). Perinatal outcome of spontaneously conceived triplet pregnancies in relation to chorionicity. Am. J. Obstet. Gynecol..

[43] Spencer J.V., Ingardia C.J., Nold C.J., Borgida A.F., Herson V.C., Egan J.F.X. (2009). Perinatal and neonatal outcomes of triplet gestations based on placental chorionicity. Am. J. Perinatol..

[44] Multiple Gestation: Biology and Epidemiology | GLOWM [Internet]. http://www.glowm.com/section-view/heading/MultipleGestation:Biologyandepidemiology/item/878.

[45] Dziadosz M., Evans M.I. (2017). Re-Thinking Elective Single Embryo Transfer: Increased Risk of Monochorionic Twinning—A Systematic Review. Fetal Diagn. Ther..

[46] Shah J.S., Roman T., Viteri O.A., Haidar Z.A., Ontiveros A., Sibai B.M. (2018). The Relationship of Assisted Reproductive Technology on Perinatal Outcomes in Triplet Gestations. Am. J. Perinatol..

[47] Weissman A., Ulanovsky I., Burke Y., Makhoul I.R., Blazer S., Drugan A. (2013). Triplet pregnancies—A three-decade perspective: Do we fare better?. Eur. J. Obstet. Gynecol. Reprod. Biol..

[48] Battin M., Wise M., DeZoete A., Stone P. (2009). Infant and perinatal outcomes of triplet pregnancy in Auckland: Better than expected?. N. Z. Med. J..

[49] Morikawa M., Cho K., Yamada T., Yamada T., Sato S., Minakami H. (2013). Clinical features and short-term outcomes of triplet pregnancies in Japan. Int. J. Gynecol. Obstet..

[50] Shah P.S., Kusuda S., Håkansson S., Reichman B., Lui K., Lehtonen L., Modi N., Vento M., Adams M., Rusconi F. (2018). Neonatal Outcomes of Very Preterm or Very Low Birth Weight Triplets. Pediatrics.

[51] Day M.C., Barton J.R., O’Brien J.M., Istwan N.B., Sibai B.M. (2005). The effect of fetal number on the development of hypertensive conditions of pregnancy. Obstet. Gynecol..

[52] Luke B., Brown M.B., Hediger M.L., Misiunas R.B., Anderson E. (2006). Perinatal and early childhood outcomes of twins versus triplets. Twin Res. Hum. Genet..

[53] Razavi A.S., Kalish R.B., Coombs S., Ragsdale E.S., Chasen S. (2017). Preterm delivery in triplet pregnancies. J. Matern. Fetal Neonatal Med..

[54] Salihu H.M., Aliyu M.H., Akintobi T.H., Pierre-Louis B.J., Kirby R.S., Alexander G.R. (2003). The impact of advanced maternal age (> or = 40 years) on birth outcomes among triplets: A population study. Arch. Gynecol. Obstet..

[55] Holcberg G., Biale Y., Lewenthal H., Insler V. (1982). Outcome of pregnancy in 31 triplet gestations. Obstet. Gynecol..

[56] Alamia V., Royek A.B., Jaekle R.K., Meyer B.A. (1998). Preliminary experience with a prospective protocol for planned vaginal delivery of triplet gestations. Am. J. Obstet. Gynecol..

[57] Adams D.M., Sholl J.S., Haney E.I., Russell T.L., Silver R.K. (1998). Perinatal outcome associated with outpatient management of triplet pregnancy. Am. J. Obstet. Gynecol..

[58] Albrecht J.L., Tomich P.G. (1996). The maternal and neonatal outcome of triplet gestations. Am. J. Obstet. Gynecol..

[59] Mastrobattista J.M., Skupski D.W., Monga M., Blanco J.D., August P. (1997). The rate of severe preeclampsia is increased in triplet as compared to twin gestations. Am. J. Perinatol..

[60] Zuppa A.A., Scorrano A., Cota F., D’Andrea V., Fracchiolla A., Romagnoli C. (2007). Neonatal outcomes in triplet pregnancies: Assisted reproduction versus spontaneous conception. J. Perinat. Med..

[61] Dudenhausen J.W., Misselwitz B., Piedvache A., Maier R.F., Weber T., Zeitlin J., Schmidt S., Martens E., Martens G., Van Reempts P. (2019). Characteristics, management and outcomes of very preterm triplets in 19 European regions. Int. J. Gynecol. Obstet..

[62] Ko H.S., Wie J.H., Choi S.K., Park I.Y., Park Y.-G., Shin J.C. (2018). Multiple birth rates of Korea and fetal/neonatal/infant mortality in multiple gestation. PLoS ONE.

[63] Mol B.W., Bergenhenegouwen L., Velzel J., Ensing S., van de Mheen L., Ravelli A.C., Kok M. (2019). Perinatal outcomes according to the mode of delivery in women with a triplet pregnancy in The Netherlands. J. Matern.-Fetal Neonatal Med..

[64] Luke B., Brown M.B. (2007). The effect of plurality and gestation on the prevention or postponement of infant mortality: 1989–1991 versus 1999–2001. Twin Res. Hum. Genet..

[65] National Collaborating Center for Women’s and Children’s Health (UK) (2011). Multiple Pregnancy: The Management of Twin and Triplet Pregnancies in the Antenatal Period [Internet].

[66] Kahn B., Lumey L., Zybert P.A., Lorenz J.M., Cleary-Goldman J., D’Alton M.E., Robinson J.N. (2003). Prospective risk of fetal death in singleton, twin, and triplet gestations: Implications for practice. Obstet. Gynecol..

[67] Unal E.R. (2015). Fetal Surveillance and Timing of Delivery for Multiples. Clin. Obstet. Gynecol..

[68] Morikawa M., Yamada T., Yamada T., Sato S., Minakami H. (2011). Contribution of twin-to-twin transfusion syndrome to preterm birth among monochorionic biamniotic and bichorionic biamniotic twin pregnancies. J. Perinat. Med..

[69] Morikawa M., Yamada T., Yamada T., Sato S., Minakami H. (2012). Prospective risk of intrauterine fetal death in monoamniotic twin pregnancies. Twin Res. Hum. Genet..

[70] Hruby E., Hajdú J., Görbe E., Hupuczi P., Papp Z. (2007). Maternal age as a risk factor in triplet pregnancy. Orvosi Hetil..

[71] Lachowska M., KO B. (2016). Respiratory Disorders and Neonatal Outcome of Triplet Pregnancies: Own Experience. Am. J. Perinatol..

[72] Cowherd R.B., Cipres D.T., Chen L., Barry O.H., Estevez S.L., Yee L.M. (2022). The Association of Twin Chorionicity with Maternal Outcomes. Am. J. Perinatol..

[73] Machtinger R., Sivan E., Maayan-Metzger A., Moran O., Kuint J., Schiff E. (2011). Perinatal, postnatal, and maternal outcome parameters of triplet pregnancies according to the planned mode of delivery: Results of a single tertiary center. J. Matern. Fetal Neonatal Med..

[74] Salihu H.M., Bagchi S., Aliyu Z.Y., Kirby R.S., Alexander G.R. (2005). Advanced maternal age and fetal growth inhibition in triplets. J. Reprod. Med..

[75] Tandberg A., Bjørge T., Nygård O., Børdahl P., Skjaerven R. (2010). Trends in incidence and mortality for triplets in Norway 1967–2006: The influence of assisted reproductive technologies. BJOG Int. J. Obstet. Gynaecol..

[76] Eddib A., Penvose-Yi J., Shelton J.A., Yeh J. (2007). Triplet gestation outcomes in relation to maternal prepregnancy body mass index and weight gain. J. Matern.-Fetal Neonatal Med..

[77] Lamb D.J., Vink J.M., Middeldorp C.M., van Beijsterveldt C.E., Haak M.C., Overbeek L.I.H., Boomsma D.I. (2012). Effects of chorionicity and zygosity on triplet birth weight. Twin Res. Hum. Genet..

[78] Nasseri F., Azhir A. (2009). The neonatal outcome in twin versus triplet and quadruplet pregnancies. J. Res. Med. Sci..

[79] Chen H.-Y., Chauhan S.P. (2019). Risk of Neonatal and Infant Mortality in Twins and Singletons by Gestational Age. Am. J. Perinatol..

